# USP7 targeting modulates anti-tumor immune response by reprogramming Tumor-associated Macrophages in Lung Cancer

**DOI:** 10.7150/thno.47137

**Published:** 2020-07-23

**Authors:** Xiaomeng Dai, Lisen Lu, Suke Deng, Jingshu Meng, Chao Wan, Jing Huang, Yajie Sun, Yan Hu, Bian Wu, Gang Wu, Jonathan F. Lovell, Honglin Jin, Kunyu Yang

**Affiliations:** 1 Cancer Center, Union Hospital, Tongji Medical College, Huazhong University of Science and Technology, Wuhan 430022, China.; 2 Department of Biomedical Engineering, University at Buffalo, State University of New York. Buffalo, New York 14260, USA.

**Keywords:** Deubiquitinating enzymes, USP7, tumor-associated macrophages, macrophages reprogramming, anti-PD-1

## Abstract

**Background:** Tumor associated macrophages (TAMs) have strong plasticity and if reprogrammed, can clear tumor cells and regulate the adaptive immune system for cancer immunotherapy. Deubiquitinating enzymes (DUBs), which can remove ubiquitin (Ub) from Ub-modified substrates, have been associated with oncogenic metabolism but are not well-known for regulating TAMs repolarization.

**Methods:** The expression of DUB related genes in macrophages (MΦs) was detected by reverse transcription-PCR. Flow cytometry and immunofluorescence were used to detect the changes of immune cells in the tumor microenvironment and spleen, including M1 (CD11b^+^F4/80^+^CD86^+^CD206^-^), and M2 (CD11b^+^F4/80^+^CD86^-^CD206^+^) MΦs, and IFN-γ^+^CD8^+^T cells. A proliferation assay was used to determine the effect of M2 MΦs treated with a USP7 inhibitor on T cell proliferation. Western blotting was used to detect the expression of USP7 and the activation of the MAPK pathway. The TGCA database was used to assess the role of USP7 in the immune microenvironment of human lung adenocarcinoma (LUAD).

**Results:** 51 DUB genes were screened and USP7 was identified as a highly expressed gene in M2 but not M1 MΦs. Specific silencing of USP7 using siRNA or USP7 inhibitors led to phenotypical and functional changes in M2 MΦs, favoring CD8^+^T cells proliferation *in vitro*. USP7 inhibitors delayed tumor growth in mice with Lewis lung carcinoma, and promoted tumor infiltration of M1 MΦs and IFN-γ^+^CD8^+^T cells. Depletion of TAMs attenuated these therapeutic effects. USP7 inhibition was shown to mediate MΦs reprogramming by activating the p38 MAPK pathway. Administration of USP7 inhibitors increased the expression of programmed cell death ligand 1 (PD-L1) in tumors, while blocking programmed cell death protein 1 (PD-1) provided an effective anti-tumor response. Clinical databases suggest that high expression of USP7 in LUAD was negatively correlated with innate and adaptive immunity.

**Conclusions:** Taken together, these results provide evidence to suggest that therapeutic approaches targeting USP7, in combination with immunotherapy, should be considered for lung cancer treatment.

## Introduction

Lung cancer is one of the leading causes of cancer-related deaths worldwide 1. In recent years, immune checkpoint blockade using agents such as programmed cell death protein 1 (PD-1) and programmed cell death ligand 1 (PD-L1) antibodies, which can revert effector T cell exhaustion, has become an established strategy in lung cancer treatment regimens 2. However, a significant portion of lung cancer patients do not benefit from these treatments, due in part to the immunosuppressive tumor microenvironment (TME) where various cells (e.g. immune cells, stromal cells, fibroblast cells and endothelial cells) can dynamically communicate with tumor cells in a variety of ways (including exosomes and microvesicles), giving rise to resistance to immunotherapy 3-6. Therefore, understanding and developing a strategy to target regulation of various cells in the TME - especially immunosuppressive cells - will not only shed new light on TME regulation but also bring new options for precision treatment of lung cancer.

Tumor-associated macrophages (TAMs) are the most abundant infiltrating inflammatory cells in the TME and are closely related to the occurrence and development of various tumors 7-9. Based on stimulation cues within the microenvironment, macrophages (MΦs) can differentiate into two major phenotypes: M1 MΦs (tumor-suppressing subtype) and M2 MΦs (tumor-promoting subtype) 10, 11. In the TME, TAMs may transform dynamically between M1 and M2 MΦs. Moreover, TAMs are often educated to polarize into M2 MΦs by cytokines, such as IL-10, IL-4, and TGF-β, which are secreted by tumor cells 12. In theory, there are three ways to reverse the tumor-promoting effect of TAMs: 1) inhibit the recruitment of TAMs, 2) clear TAMs from tumor tissues, and 3) reprogram TAMs from M2 to M1 phenotype 12-15. However, reduction of TAMs could also result in a significant decrease in the number of M1 MΦs, which have anti-tumor effect. This makes reprogramming TAMs from M2 to M1 MΦs an appealing treatment strategy 16, 17. Although oligonucleic acid drugs (miRNA or siRNA) and Toll-like receptor agonists have been shown to induce reprogramming of TAMs in animal experiments, their effect in clinical studies is limited, and they have possible side effects such as systemic inflammatory response and hematological toxicity 18. Therefore, there is a need to identify new TAM reprogramming agents for precision cancer medicine.

Ubiquitination is one of the most important pathways of protein degradation. Following ubiquitin modification, proteins are recognized and degraded by proteasomes 19. Correspondingly, there is a complex deubiquitinating enzyme (DUB) system which can regulate the stability and function of proteins by removing ubiquitin chains 20. DUBs regulate the metabolic level of substrate proteins by cleaving monoubiquitin or polyubiquitin molecules from them, thereby regulating a variety of cellular activities, including gene transcription, cell cycle, DNA repair damage, tumorigenesis, and the inflammatory immune response 20-23. Inhibitors of the DUB proteins USP1, USP4, USP7, USP14, and USP33 have shown therapeutic effects in prostate cancer, lung cancer, breast cancer, and blood malignancies 24-28. In addition, USP4, USP7, USP13, and USP19 have been reported to stabilize the anti-inflammatory receptor or the release of anti-inflammatory cytokines 27, 29, 30. USP24 has been reported to promote the expression of IL-6 in TAMs 31. However, the relationship between DUBs and TAM reprogramming is currently not well understood. Exploring the role of DUBs in M1 and M2 MΦs will provide additional molecular targets for clinical immunotherapy.

In this study, the expression of 51 common genes related to DUBs was examined in M1 and M2 MΦs induced from the ANA-1 murine MΦ cell line. The difference in expression levels between M1 and M2 MΦs was the greatest for USP7. USP7 is a cysteine protease, and an important member of the DUB family 32. It is also the first deubiquitinating enzyme that has been specifically inhibited by drugs with good therapeutic effect 33-35. Related studies have shown that USP7 mainly plays the role of an oncoprotein by regulating the MDM2-P53 axis in various tumors 36, 37. At the same time, USP7 can stabilize the expression of Foxp3 to maintain the function of regulatory T (Treg) cells, and it also regulates the activation of NLRP3 inflammasome 38, 39. To the best of our knowledge, the effect of USP7 on TAM regulation has not been reported. Here, we investigated the role of USP7 in TAMs and how targeting USP7 can affect immune checkpoint blockade strategy for lung cancer. The results of this study show that targeting USP7 can reverse the immunosuppressive effects of TME and improve the efficiency of lung cancer immunotherapy.

## Methods

### Cell Cultures

Mouse Lewis cells and ANA-1 cells were obtained from the China Center for Type Culture Collection (Wuhan, China). Cells were grown in Dulbecco's Modified Eagle's Medium (DMEM) (Gibco, Grand Island, NY, USA) containing 10% Fetal Bovine Serum (FBS) (Gibco, Grand Island, NY, USA) and 1% penicillin/streptomycin solution.

### Reagents

P5091, SB203580, SP600125, and U0126-EtOH were purchased form Selleck. HBX-19818 and GNE-6776 were purchased from MCE. CFSE, LPS, CCK-8 reagent, NMP, Tween-80 and PEG400 were purchased from Biosharp (Hefei, China). Clodronate liposomes were purchased from Biolegend. PD-1 antibody was purchased from Invitrogen.

### Generation and Activation of Mouse BMDMs

Mouse bone marrow-derived macrophages (BMDMs) were generated as described previously 40. BMDMs were cultured with 20 ng/mL IL-4 (214-14-20) and 20 ng/mL IL-13 (210-13-10), to be polarized into IL-4/13 M2 MΦs; 50 ng/mL IL-10 (210-10-10), to be polarized into IL-10 M2 MΦs; or 50 ng/mL LPS and 20 ng/mL IFN-γ (315-05-20), to be polarized into M1 MΦs. All cytokines were purchased from PeproTech (Rocky Hill, NJ, USA).

### T-Cell Proliferation Assay

CD8^+^T cells were isolated from the spleens of C57BL/6 mice, following the MojoSort™ Mouse CD8^+^T Cell Isolation Kit protocol (480008, Biolegend). M2 MΦs induced by IL-4 and IL-13 were treated with P5091 for 24 h, and the conditioned medium was collected and filtered through a 0.22 μm filter. To determine the detailed MAPK pathways mediating P5091-treated M2 MΦs function, IL-4/13-BMDMs were pre-treated with 10 μM SB203580 (p38 MAPK-selective inhibitor), 10 μM SP600125 (JNK- selective inhibitor), 10 μM U0126-EtOH (ERK-selective inhibitor) for 2 h, the cells were stimulated with 10 μM P5091 for 24 h, and the conditioned medium was collected. Carboxyfluorescein succinimidyl ester (CFSE)-labeled CD8^+^T cells were cultured with IL-2 (20 ng/ml) and stimulated with Dynabeads® Mouse T-Activator CD3/CD28 (11452D, Life Technologies) in the conditioned medium for 4 days. Finally, the proliferation of CD8^+^T cells was detected by flow cytometry.

### Real-time Quantitative PCR

Total RNA was harvested from cells using MicroElute Total RNA Kit R6831-01 (Omega Bio-tek, Norcross, GA, USA) and reverse-transcribed into cDNA using HiScript III RT SuperMix (+ gDNA wiper) (Vazyme, Nanjing, China). The cDNA was amplified using the AceQ® Universal SYBR qPCR Master Mix (Vazyme, Nanjing, China) on a StepOnePlus Real-Time PCR System (Thermo Fisher Scientific, Waltham, MA, USA). Primer sequences are shown in Supplementary Table S1. All primers were synthesized by Sangon Biotech Co., Ltd (Shanghai).

### Transfections

BMDMs were seeded in 6-well-plates (2×10^5^ cells per well). After 24 h, they were transfected with either small interfering RNA (siRNA) against USP7 or negative control (NC) siRNA using Lipofectamine RNAiMAX (Invitrogen, Carlsbad, CA, USA). Cells were harvested after 48 h and processed for western blotting and flow cytometry. USP7 siRNA (sc-77373) and NC siRNA (sc-37007) were purchased from Santa Cruz Biotechnology, Inc.

### Western Blotting

Cell lysate was prepared for western blotting by lysing cells with RIPA lysis buffer, followed by SDS-PAGE separation and transfer to PVDF membrane. After blocking the membranes for 1 h at room temperature in 5% skim milk powder dissolved in Tris-buffered saline containing 5% Tween-20 (TBST), membranes were incubated overnight at 4 °C with the corresponding antibodies. Then, membranes were washed and incubated with secondary antibodies prior to detection. They were developed using NcmECL Ultra (P10100, NCM Biotech) and imaged. The specific antibodies that were used are shown in Supplementary Table S2.

### Flow Cytometry

For cell-surface analysis, cells were stained with anti-mouse Zombie Violet™ Fixable Viability Kit (423114), Zombie NIR™ Fixable Viability Kit (423106), CD45 (103114), CD11b (101205), CD86 (105012), CD4 (100408), CD8a (100752), Gr-1 (108411), and PD-L1 (124311, 124308) in recommended antibody concentrations and incubated at 4 °C for 30 min. For the T-cell intracellular IFN-γ (505808) cytokine staining, cells were fixed and permeabilized after stimulation with Phorbol 12-myristate 13-acetate (PMA) (ab120297, Abcam, 100 ng/mL), Monensin sodium salt (ab120499, Abcam, 1 ug/mL), and Ionomycin calcium salt (5608212, PeproTech, 100 ng/mL) for 6 h. For the CD206 (141706) and Foxp3 (126408) staining, cells were also fixed and permeabilized. All flow cytometry antibodies were purchased from Biolegend (San Diego, CA, USA).

### Gain of Tumor-infiltrating Immunocytes

Obtaining tumor infiltrating immune cells from tumor tissue was performed as previously described 40.

### Mouse Tumor Models and Treatment

Female C57BL/6 mice (6-week-old) were purchased from HBCDC (Wuhan, China). All mice were kept in accordance with protocols that had been approved by the Hubei Provincial Animal Care and Use Committee, as well as the guidelines of the Animal Experimentation Ethics Committee of the Huazhong University of Science and Technology (HUST, Wuhan, China). 50 μL PBS containing 1×10^6^ Lewis cells was subcutaneously injected into the right flank of female C57BL/6 mice to develop the subcutaneous tumor bearing model. The mice were divided randomly into different groups seven days later.

P5091 (40 mg/kg, i.p.), dissolved in a solution (4% NMP, 3% Tween-80 and 20% PEG400 in Milli-Q water) and Vehicle (100 μL, i.p.) were injected once a day for 12 days, respectively. Clodronate liposomes were applied for MΦ depletion in the dose of 150 μL per mouse for on the first day, followed by 100 μL per mouse every three days for a total of four times. Treatments with anti-PD-1 (10 mg/kg, i.p.) were conducted every other day, for a total of four times. Tumor volumes were calculated according to the modified ellipsoidal formula: V = 1/2 (length × width^2^).

### Tissue Multicolor Immunofluorescent Staining

Tissue multicolor immunofluorescent staining was performed by Opal^TM^ 7-Color Manual IHC Kit (NEL811001KT, Perkinelmer). Tumor tissues were fixed and embedded in paraffin and sliced with a slicer. The slices are routinely dewaxed and hydrated. Tris-EDTA Buffer solution for antigen repairing, 3% H_2_O_2_ for inactivation of endogenous peroxidase, and normal goat serum for blocking. The slides were then incubated with CD8 (A02236-1), CD86 (ab213044), CD206 (ab64693), F4/80 (ab6640), and IFN-γ (ab9657) overnight. Next, they were incubated with HRP labelled goat anti-rabbit / mouse secondary antibody in combination with DAPI for 1 h at room temperature. Finally, tissue immunofluorescence was analyzed using PE Vectra (Perkinelmer). CD8 antibody was purchased from BOSTER Biological Technology Co., Ltd (Wuhan, China). The antibodies against CD86, CD206, F4/80, and IFN-γ were purchased from Abcam.

### TAMs Sorting and RNA Sequencing

TAMs were sorted from tumor tissues of mice treated with either the Vehicle or P5091 by flow cytometry. The sorted TAMs were washed twice with PBS, centrifuged for 5 min at 500 g, and the supernatant was discarded. The cell precipitate was quickly frozen at -80 °C. Then, the MΦs were sent to Beijing Novogene Technology Co., Ltd for RNA sequencing.

### Tissue Cytokine Detection

Cytokines IFN-γ, TNF-α, IL-2, IL-5, and IL-6 in tumor tissues extracted from either the Control group or the P5091 group were detected by flow cytometry according to LEGENDplex^TM^ Multi-Analyte Flow Assay Kit (740750, Biolegend).

### TCGA Database Analysis

The original data from RNA-seq of lung adenocarcinoma were obtained from TCGA database (https://gdc.nci.nih.gov/). Data analysis was based on R×64.3.5.3 software, and the data of USP7 differential expression between normal tissue and lung adenocarcinoma tissue were analyzed according to edgeR package in R language. To extract the immune-related pathways from GO.db and biomaRt packets for Gene set variation analysis (GSVA). Referring to the seven gene sets related to T cell immunity and inflammatory immunity previously reported by Achim Rody and other researchers 41, the correlation between these seven gene sets and the USP7 expression was analyzed by GSVA.

### Statistical Analysis

We used the unpaired two-tailed Student's t-test to compare the differences between the two groups of measurements, while the survival rates were evaluated with the Kaplan-Meier test using the Graphad Prism 6 software. Repeated measurements of tumor volume growth were compared using One-Way ANOVA analysis of variance. Flow cytometry data were analyzed by FlowJo.10. Significant differences between the groups are indicated by *P <0.05, **P < 0.01, ***P < 0.001.

## Results

### Targeting USP7 inhibits the M2 phenotype and function of murine MΦs *in vitro*

**Figure 1:**
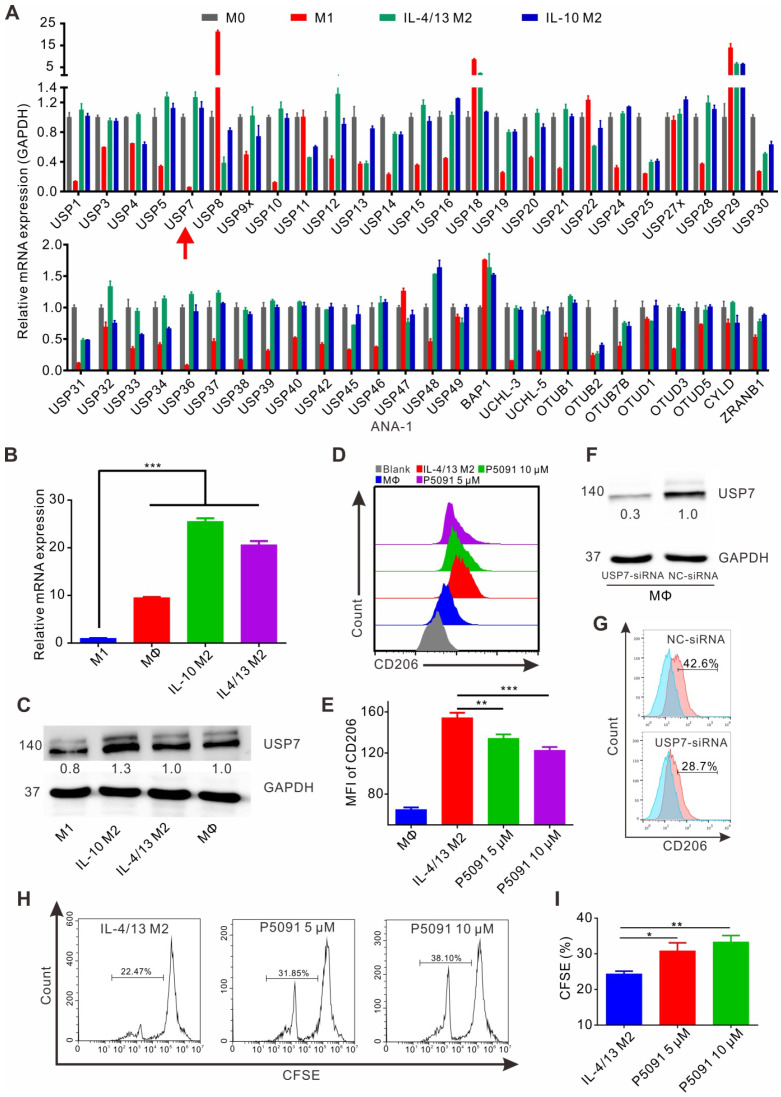
** Targeting USP7 inhibits murine M2 phenotype and function* in vitro***.** (A)** The mRNA expression of common genes related to DUBs was detected by RT-PCR in M0, M1 (LPS/IFN-γ M1), and M2 (IL-4/13 M2 and IL-10 M2) induced from ANA-1. (**B**) The mRNA expression of USP7 in MΦs, M1, and M2 induced from BMDMs was detected by RT-PCR. (**C**) Western blotting showing the expression of USP7 in MΦ, M1, and M2 induced from BMDMs. (**D-E**) Flow cytometry analyses of the expression of CD206 in IL-4/13-induced BMDM M2 cells, which had been treated with P5091 (5 μM or 10 μM) for 24 h. Data are presented as the mean ± SEM (n = 3). (**F**) Detection of the expression of USP7 in BMDMs, which were transfected with either NC- or USP7-siRNA by western blotting. (**G**) The expression of CD206 in BMDMs of NC-siRNA and USP7-siRNA group was detected by flow cytometry. (**H-I**) Flow cytometry analyses of CFSE expression on the surface of CD8^+^T cells in the presence of conditioned medium from either DMSO- or P5091- (5 μM or 10 μM) treated IL-4/13-induced BMDM M2 cells. Data are presented as the mean ± SEM (n = 3).

Despite TAMs being closely related to the occurrence and development of lung cancer 42, there are only limited reports on the involvement of DUBs in regulation of their function. Having this in mind, the expression of common genes related to DUBs was assessed by reverse transcription-PCR (RT-PCR) in M1 and M2 MΦs induced from the ANA-1 murine MΦ cell line. As shown in **Figure 1A**, mRNA levels of the 51 DUB genes exhibited different expression patterns, among which the differential expression of USP7 between M2 (IL-4/13 M2 and IL-10 M2) and M1 MΦs was the most obvious DUB, with the highest M2/M1 ratio of 21.7 (IL-4/13 M2/M1) and 19.2 (IL-10 M2/M1). The expression of the translated USP7 protein in M2 MΦs (IL-4/13 M2 and IL-10 M2; induced from ANA-1) was confirmed to significantly higher than in M1 MΦs (**Figure S1**). Moreover, on account of it being the first DUB to be specifically inhibited by small molecule inhibitors, we were particularly interested in USP7. Next, we further verified the expression of USP7 in M2 and M1 MΦs induced from BMDMs. As shown in **Figure 1B-C**, the expression of USP7 mRNA and the corresponding protein in M2 MΦs (IL-4/13 M2 and IL-10 M2) was significantly higher than that in M1 MΦs. Altogether, these data confirm the differential expression of USP7 between M2 and M1 MΦs. Motivated by this finding, we explored whether targeted inhibition of USP7 can selectively kill M2 MΦs. We used specific inhibitors of USP7 (P5091, HBX19818, GNE-6776) to treat M1, IL-4/13 M2, and IL-10 M2 MΦs derived from ANA-1. These three inhibitors selectively bound the USP7 active site and inhibited its activity, but not its expression 35, 43. Moreover, we further verified that treatment of ANA-1 cells with three inhibitors (P5091, HBX19818, GNE-6776, 10 μM) did not alter USP7 expression (**Figure S2**). No significant selectivity was observed between M2 and M1 killing for any of the three inhibitors (**Figure S3A-C**) under the tested conditions. At the same time, we further verified the non-selective killing effect of P5091 on M2 and M1 MΦs induced from BMDMs (**Figure S3D**).

Next, we examined the effects of targeted inhibition of USP7 on the phenotypical and functional changes of M2 MΦs. According to **Figure S4A-B**, all three inhibitors (P5091, HBX19818, GNE-6776), in the concentration range (0-10 μM) that had negligible toxicity to tested cells, significantly reduced the expression of CD206 (M2-related marker) in IL-4/13 M2 and IL-10 M2 MΦs induced from ANA-1. Using IL-4/13 M2 MΦs induced from BMDMs, we further validated that both P5091 (**Figure 1D-E**) and GNE-6776 significantly reduced the expression of CD206, while HBX19818 had no such effect at a concentration of 10 μM (**Figure S4C**) - possibly due to the relatively weaker UPS7 inhibitory activity of HBX19818 (IC50: 28.1 μM) compared to that of P5091 (4.2 μM). Additionally, targeting USP7 with P5091 had no obvious effect on the expression of CD86 (M1-related marker) in IL-4/13 M2 and IL-10 M2 MΦs induced from ANA-1, nor in primary IL-4/13 M2 MΦs (**Figure S5**). To further clarify the regulatory role of USP7 in phenotypical changes of M2 MΦs, we used siRNA to knock down the expression of USP7 in BMDMs. **Figure 1F-G** showed that, compared to negative control (NC) group, the expression of CD206 was significantly decreased in USP7-siRNA group. Finally, we detected the effect of the conditioned medium of M2 MΦs, which had been treated with USP7 inhibitor P5091, on the proliferation of CD8^+^T cells by CFSE assay. We found that the proliferation rates of CD8^+^T cells in P5091 (5 μM and 10 μM) groups were significantly higher than that of IL-4/13 M2 group, which further indicated that targeting USP7 could inhibit the function of M2 MΦs (**Figure 1H-I**). In summary, targeted inhibition of USP7 can alter the phenotype and inhibit the function of mouse M2 MΦs.

### Targeting USP7 inhibits tumor growth and induces anti-tumor immunity *in vivo*

**Figure 2:**
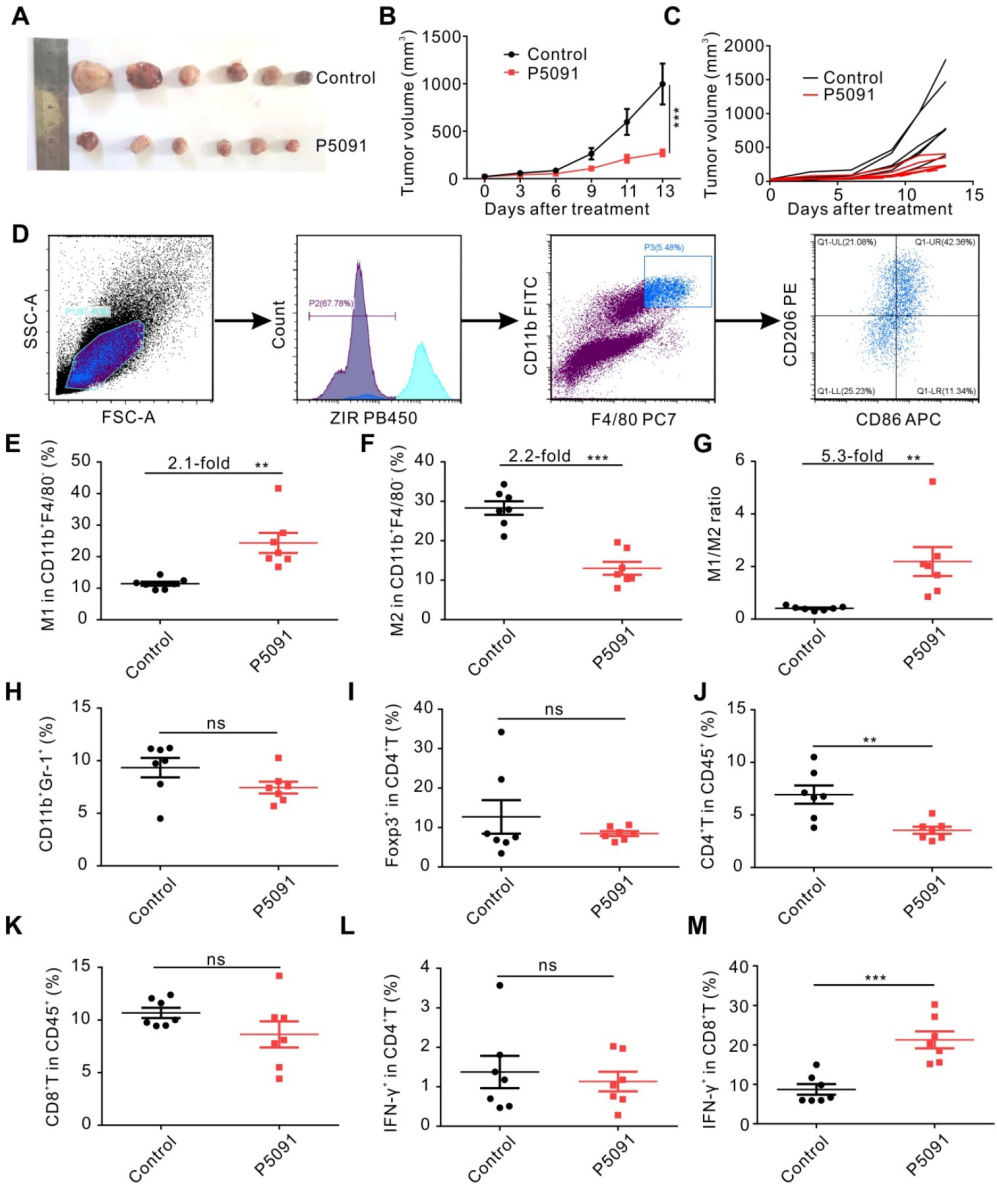
** Targeting USP7 inhibits tumor growth and induces local anti-tumor immunity *in vivo*.** (**A**) Harvested and photographed tumors in the P5091 and the Control group. (**B**) Lewis tumor growth following P5091 or Vehicle treatment *in vivo.* Data are presented as the mean ± SEM (n = 6 per group). (**C**) Spider diagram of the tumor volume growth in each mouse from the P5091 group and the Control group. (**D**) Gating strategy for detection of the TAMs by flow cytometry. (**E-G**) Proportions of M1 (**E**) and M2 (**F**), and M1/M2 ratio (**G**) in the TME of the P5091 group and the Control group. (**H-M**) Percentages of MDSC (**H**), Treg (**I**), CD4^+^T (**J**), CD8^+^T (**K**), Th1 (**L**), and CTLs (**M**) within the TME in each group. Data are presented as the mean ± SEM (n = 7) for (**E-M**).

We explored the effects of targeting USP7 on TAMs and anti-tumor immunity *in vivo*, using a subcutaneous Lewis tumor-bearing mouse model. Intraperitoneal injection of P5091 (40 mg/kg) effectively inhibited tumor growth and resulted in an average inhibition rate of 73%, calculated by tumor volume (**Figure 2A-C**), and no obvious changes in mice body weight compared to the control group (**Figure S6**). To further examine the effects of intraperitoneal injection of P5091 on innate and adaptive anti-tumor immunity, on day 13 after the P5091 treatment we detected MΦs, myeloid-derived suppressor cell (MDSCs), regulatory T (Treg) cells, help T lymphocyte 1 (Th1) cells, and cytotoxic T lymphocytes (CTLs) by flow cytometry. The gating strategies for the detection of the ratio of MΦs and T cells in TME are shown in **Figure 2D** and **Figure S7**, respectively. According to **Figure 2E-G**, the proportion of M1 MΦs (ZIR^-^CD11b^+^F4/80^+^CD86^+^CD206^-^) and the ratio of M1/M2 in P5091 group were 2.1 and 5.3 times higher than those in the control group, respectively; while the proportion of M2 MΦs (CD11b^+^F4/80^+^CD86^-^CD206^+^) had a significant reduction of 2.2 times. These results indicated that P5091 can effectively reprogram TAMs from M2 to M1 phenotype. On the other hand, there was no significant change in the ratio of MDSCs (ZIR^-^CD11b^+^Gr1^+^) and Tregs (ZIR^-^CD45^+^CD4^+^Foxp3^+^) in TME after the P5091 treatment (**Figure 2H-I**). Furthermore, we detected changes of anti-tumor immune responses mediated by T cells in the TME. Compared to the control group, the proportion of CD4^+^T cells decreased significantly (**Figure 2J**), while the proportions of CD8^+^T cells and Th1 (ZIR^-^CD45^+^CD4^+^IFN-γ^+^) cells did not (**Figure 2K-L**). The proportion of CTLs (ZIR^-^CD45^+^CD8^+^IFN-γ^+^) dramatically increased in the TME after the P5091 treatment (**Figure 2M**).

**Figure 3:**
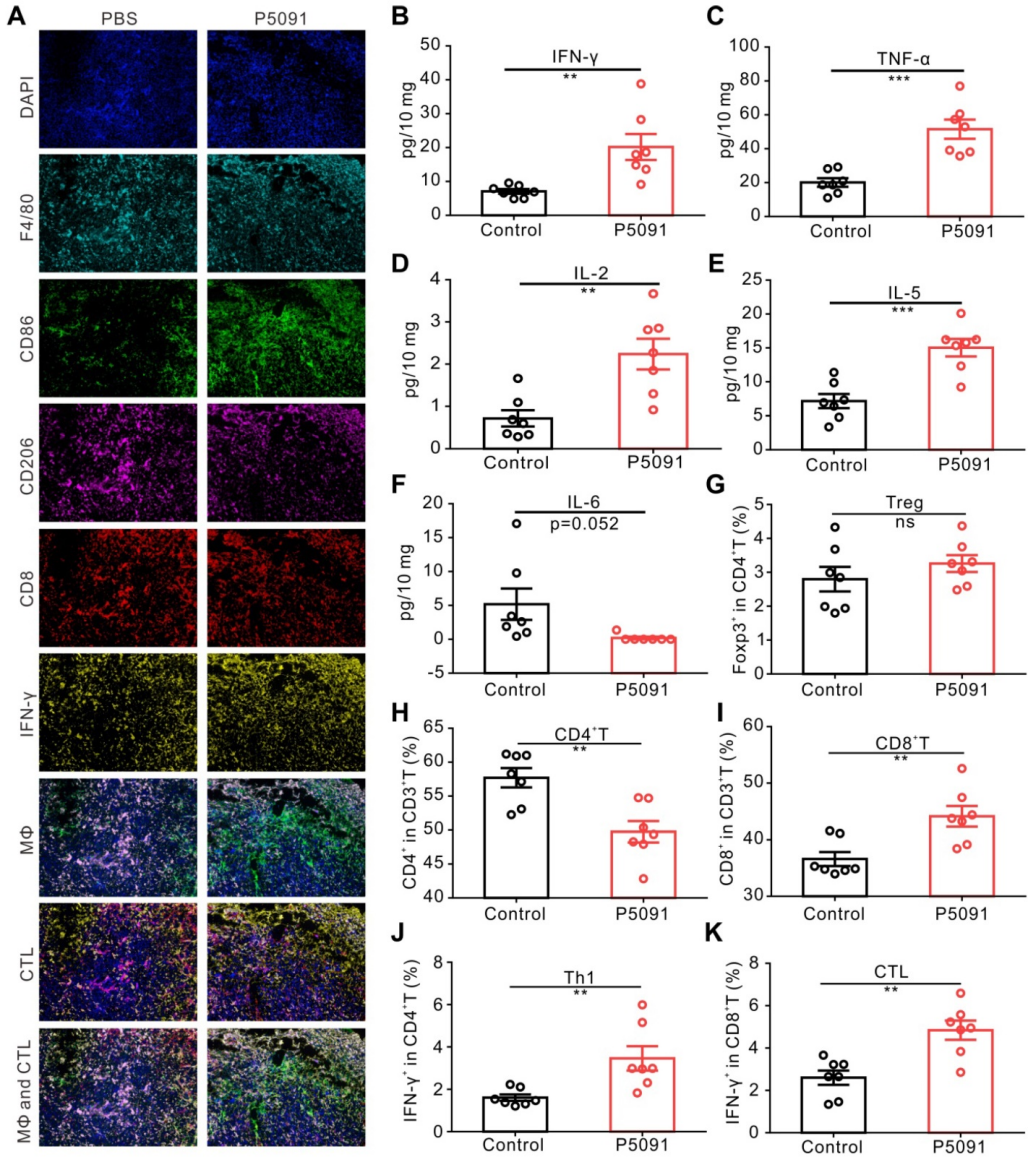
** Targeting USP7 activates local and systemic anti-tumor immunity.** (**A**) Multicolor immunofluorescence detection of TAMs and CTLs in the TME of the P5091 group and the Control group. (**B-F**) Cytokines IFN-γ (**B**), TNF-α (**C**), IL-2 (**D**), IL-5 (**E**), and IL-6 (**F**) in the TME of each group were detected by Mul-Analyte Flow Assay Kit. (**G-K**) Proportion of Treg (**G**), CD4^+^T (**H**), CD8^+^T (**I**), Th1 (**J**), and CTLs (**K**) in the spleen of each group. Data are presented as the mean ± SEM (n = 7) for (**B-K**).

To further confirm the observed changes of MΦs and CTLs, multicolor immunofluorescence detection was performed, in which tumor tissues were simultaneously labeled with the antibodies of DAPI, F4/80, CD86, CD206, CD8, and IFN-γ. The number of M1 MΦs (DAPI^+^F4/80^+^CD86^+^) and CTLs (DAPI^+^CD8^+^IFN-γ^+^) in the P5091 group was significantly higher than that in the control group, while the number of M2 MΦs (DAPI^+^F4/80^+^CD206^-^) was significantly lower than that in the control group (**Figure 3A**); which was consistent with the results of flow cytometry. Additionally, changes in the content of cytokines in the TME were assessed by Mul-Analyte Flow Assay Kit. As shown in **Figure 3B-F**, compared to the control group, the content of anti-tumor immunity-related cytokines IFN-γ, TNF-a, IL-2, and IL-5 increased, while IL-6, related to inhibiting anti-tumor immunity, decreased in the TME. Altogether, these results suggest that targeting USP7 can significantly promote polarization of TAMs into M1 MΦs and activate the anti-tumor immune responses mediated by CTLs in the TME *in vivo*.

To explore whether targeting USP7 can activate systemic adaptive anti-tumor immunity, we examined the population of CD4^+^T cells, CD8^+^T cells, Th1 cells, and CTLs in the spleen. The results showed that compared to the control group, the proportion of CD4^+^T cells decreased significantly, whereas the proportion of CD8^+^T cells, Th1 cells, and CTLs increased effectively, while there was no significant change in the proportion of Treg cells in the spleen of the P5091 group (**Figure 3G-K**). Therefore, targeting USP7 can significantly activate systemic anti-tumor immune response.

### Mechanism of targeting USP7-mediated anti-tumor effect

**Figure 4:**
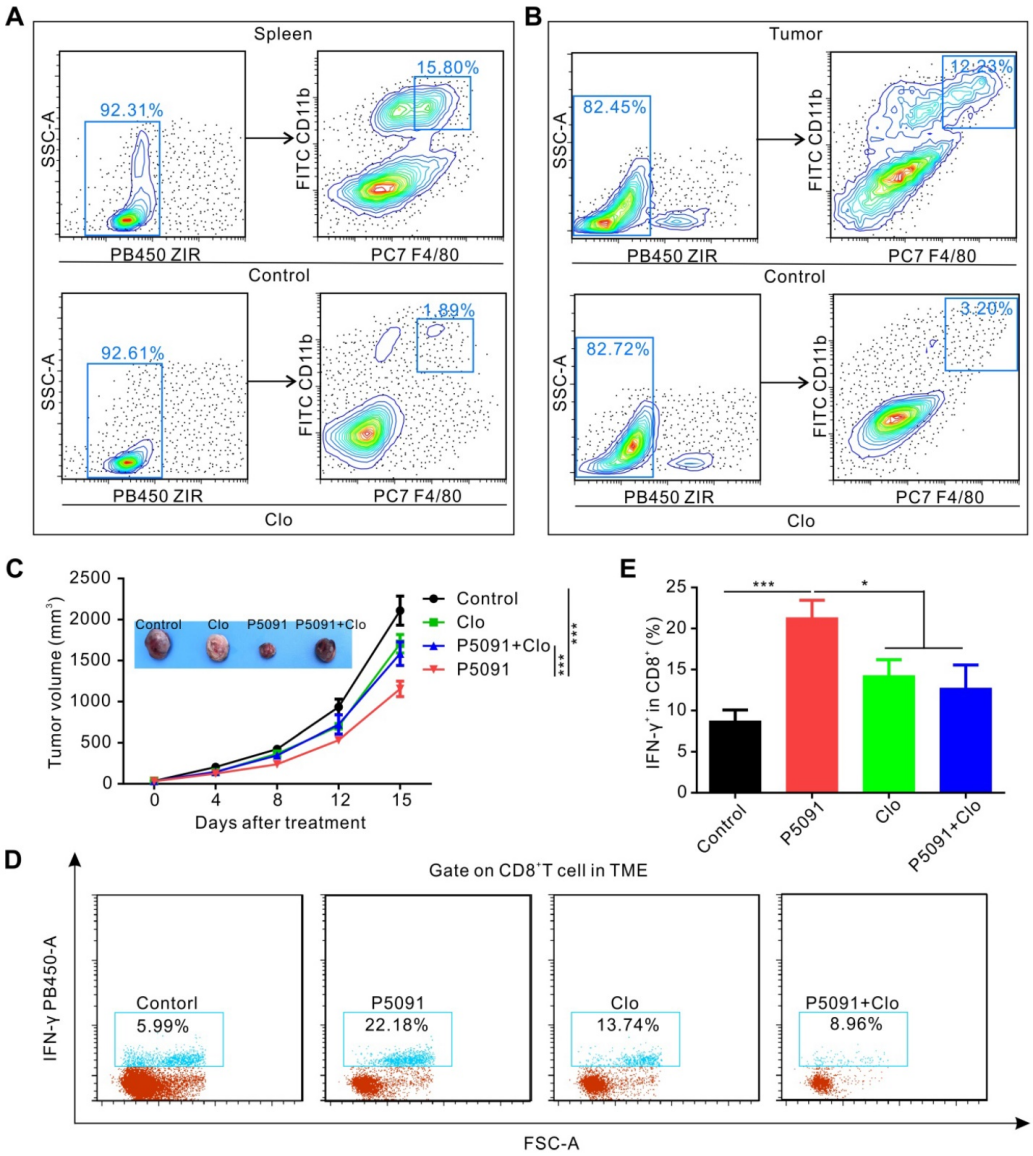
** P5091-mediated tumor inhibition effect and CTL activation are MΦ-dependent.** (**A-B**) Verification of the efficiency of clodronate liposome (Clo) in depleting MΦs of the spleen (**A**) and the TME (**B**). (**C**) Tumor growth monitored for 16 days in Lewis tumor-bearing mice after various indicated treatments. (**D**) Representative dot plots showing the proportion of CTLs in the TME of each group. (**E**) Summarized data from the dot plots showing the percentage of CTLs in the TME. Data are presented as the mean ± SEM (n = 7) for (**C, E**).

To determine whether targeting USP7-mediated anti-tumor immune response is dependent on MΦs, we used clodronate liposomes (Clo) to deplete MΦs from mice. As expected, the clearance efficiency of Clo on splenic MΦs and TAMs reached about 88% and 74%, respectively (**Figure 4A-B**). As depicted in** Figure 4C**, MΦ depletion by Clo significantly attenuated the inhibitory effect of P5091 in the Lewis tumor-bearing mouse model. Moreover, we found that MΦ depletion also disrupted the effects of CTL activation induced by P5091 in the TME (**Figure 4D-E**). Taken together, these data suggest that P5091-mediated anti-tumor effect and CTL activation were MΦ-dependent.

**Figure 5:**
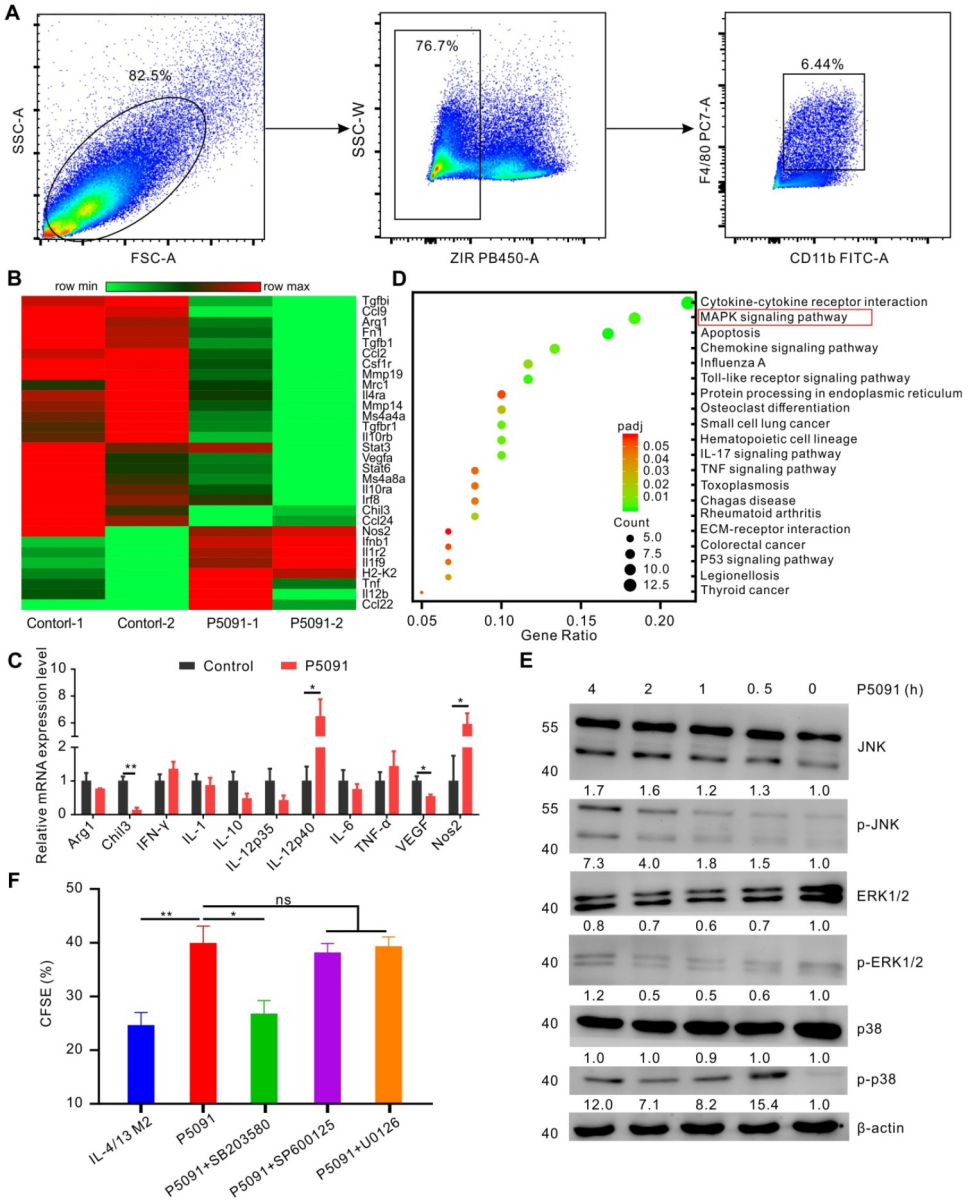
** Targeting USP7 can activate the p38 MAPK pathway to reprogram TAMs.** (**A**) The strategy for sorting TAMs in TME by flow cytometry. (**B**) Heat maps illustrating the differentially expressed M1- and M2-related genes in TAMs between the P5091 group and the Control group based on the results of RNA sequencing. (**C**) RT-PCR further verifying the differentially expressed genes of sorted TAMs in each group. Data are presented as the mean ± SEM (n = 3). (**D**) KEGG analysis identifying 20 most obviously enriched pathways based on the differentially expressed genes of the two groups. (**E**) Western blotting detection of the expression of JNK, p-JNK, ERK1/2, p-ERK1/2, p38, p-p38, and β-actin in IL-4/13-BMDM M2 cells treated with P5091 (10 µM) at the indicated time points. (**F**) Flow cytometry analysis of CFSE expression on the surface of CD8^+^T cells in the presence of conditioned medium of IL-4/13-induced BMDM M2 cells from various indicated treatments. Treatments indicated: DMSO stimulation, P5091 (10 µM) stimulation, P5091 (10 µM) stimulation in the presence of inhibitors of p38 (SB203580, 10 µM), JNK (SP600125, 10 µM), Erk1/2 (U0126-EtOH, 10 µM). Data are presented as the mean ± SEM (n = 4).

To delineate the underlying molecular mechanism of the targeted inhibition of USP7-mediated remodeling of TAMs, we performed RNA sequencing analysis of TAMs, sorted from the control group and P5091-treated groups by flow cytometry (**Figure 5A**). Compared to the control group, M2-related genes (Arg1, Chi3, VEGF, *etc*.) were effectively down-regulated, while M1-related genes (Nos2, IL-12, *etc*.) were significantly up-regulated after the P5091 treatment (**Figure 5B**). Quantitative RT-PCR analysis further verified that P5091-treated TAMs expressed greater mRNA levels of M1-associated IL-12p40 and Nos2, as well as lower levels of M2-associated Chil3 and VEGF (**Figure 5C**). To further evaluate the effect of P5091 on reprogramming of TAMs, we used the Kyoto Encyclopedia of Genes and Genomes (KEGG) to identify the enriched canonical signaling pathway in TAMs. As shown in **Figure 5D**, the M1-related MAPK signaling pathway was significantly enriched in the P5091-treated group compared to the control group. Western blotting further confirmed that the phosphorylation levels of JNK, ERK, and p38 proteins, which are related to the MAPK pathway, were effectively increased in P5091-treated IL-4/13 M2 MΦs induced from both BMDMs and ANA-1 (**Figure 5E and S8**). To further explore whether the reprogramming of macrophages by USP7 inhibitor was dependent on its regulation on MAPK pathways, we performed related rescue experiments by using inhibitors of Erk1/2 (U0126- EtOH), p38 (SB203580) and JNK (SP600125) 44. As shown in **Figure 5F and Figure S9**, compared with IL-4/13 M2 group, P5091-induced CD8^+^T proliferation was significantly blocked by SB203580, but not U0126-EtOH or SP600125, suggesting that *in vitro,* targeted inhibition of USP7 decreases the function of mouse M2 MΦs mainly through activation of the p38 MAPK pathway. Collectively, targeted inhibition of USP7 activated p38 MAPK pathway that resulted in TAMs reprograming.

### Combined blockade of USP7 and anti-PD-1 exerts synergistic anti-tumor effects *in vivo*

**Figure 6:**
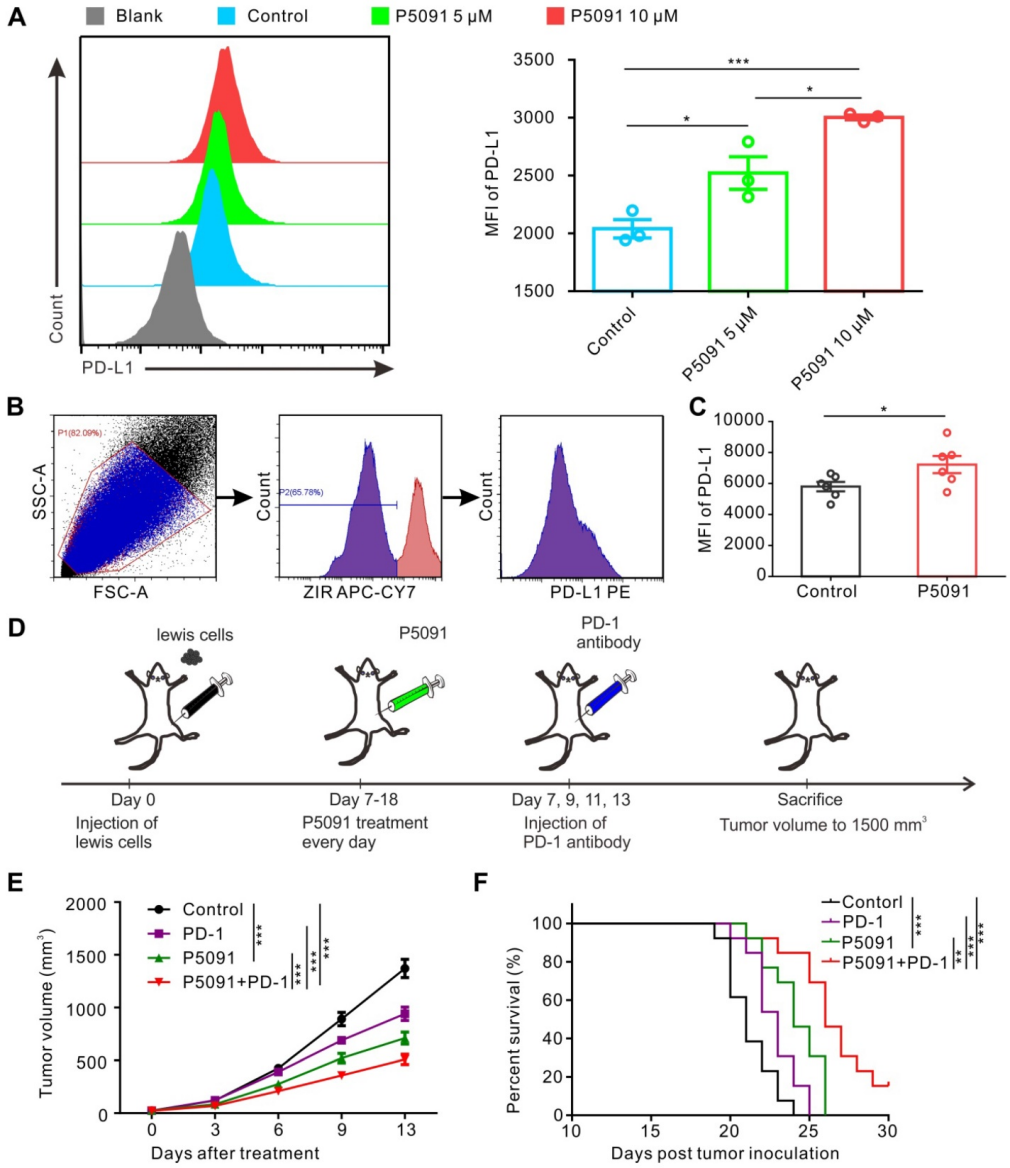
** Combined blockade of USP7 and PD-1 exerts synergistic anti-tumor effect *in vivo*.** (**A**) Flow cytometry detection of PD-L1 expression in Lewis cells after the P5091 (5 µM or 10 µM) treatment. Data are presented as the mean ± SEM (n = 3). (**B**) Flow cytometry gating strategy for the detection of PD-L1 expression in the Lewis TME. (**C**) Quantification of PD-L1 expression in the Lewis TME (ZIR- living cells) after the P5091 treatment by flow cytometry. Data are presented as the mean ± SEM (n = 6). (**D**) Treatment schedule of the combination of P5091 and PD-1 antibody for the Lewis tumor-bearing mice. (**E**) Mice bearing Lewis tumors were treated with P5091 and/or anti-PD-1 for 14 days (n = 13). (**F**) Kaplan-Meier survival plot showing survival of mice after the indicated treatments (n = 13).

Related studies have reported that promoting polarization of TAMs from M2 into M1 MΦs tends to increase the expression of PD-L1 40, 45. Therefore, we monitored PD-L1 expression in BMDMs after P5091 treatment, but no significant changes were detected (data not shown). On the other hand, strikingly, P5091 significantly increased the expression of PD-L1 in Lewis tumor cells in a concentration-dependent manner (**Figure 6A**). Additionally, we monitored the expression of PD-L1 in the living cells of TME after the P5091 treatment in the Lewis model mice. According to **Figure 6B-C**, the expression of PD-L1 in the TME of the P5091 group was significantly higher than that in the control group. The results of tumor histochemical staining of PD-L1 in the P5091 and the control group were in line with those of flow cytometry (**Figure S10**). These results suggested that targeted inhibition of USP7 *in vivo* can increase the expression of PD-L1 in the TME. Therefore, we explored whether the combination of P5091 and anti-PD-1 exerted synergistic tumor inhibition *in vivo*. In this experiment, mice were divided into a control group, a P5091 treatment group, an anti-PD-1 group, and a P5091 + anti-PD-1 group (**Figure 6D**). As shown in **Figure 6E**, treatment with P5091 + anti-PD-1 significantly delayed the Lewis subcutaneous tumor growth compared to the other three groups. At the same time, Kaplan-Meier survival analysis showed that the P5091 + anti-PD-1 group had the longest survival time compared to the other three groups (**Figure 6F**). In summary, targeted inhibition of USP7 combined with anti-PD-1 can exert a synergistic effect on the Lewis subcutaneous tumor.

### High expression of USP7 in LUAD is negatively associated with anti-tumor immunity in TCGA

**Figure 7:**
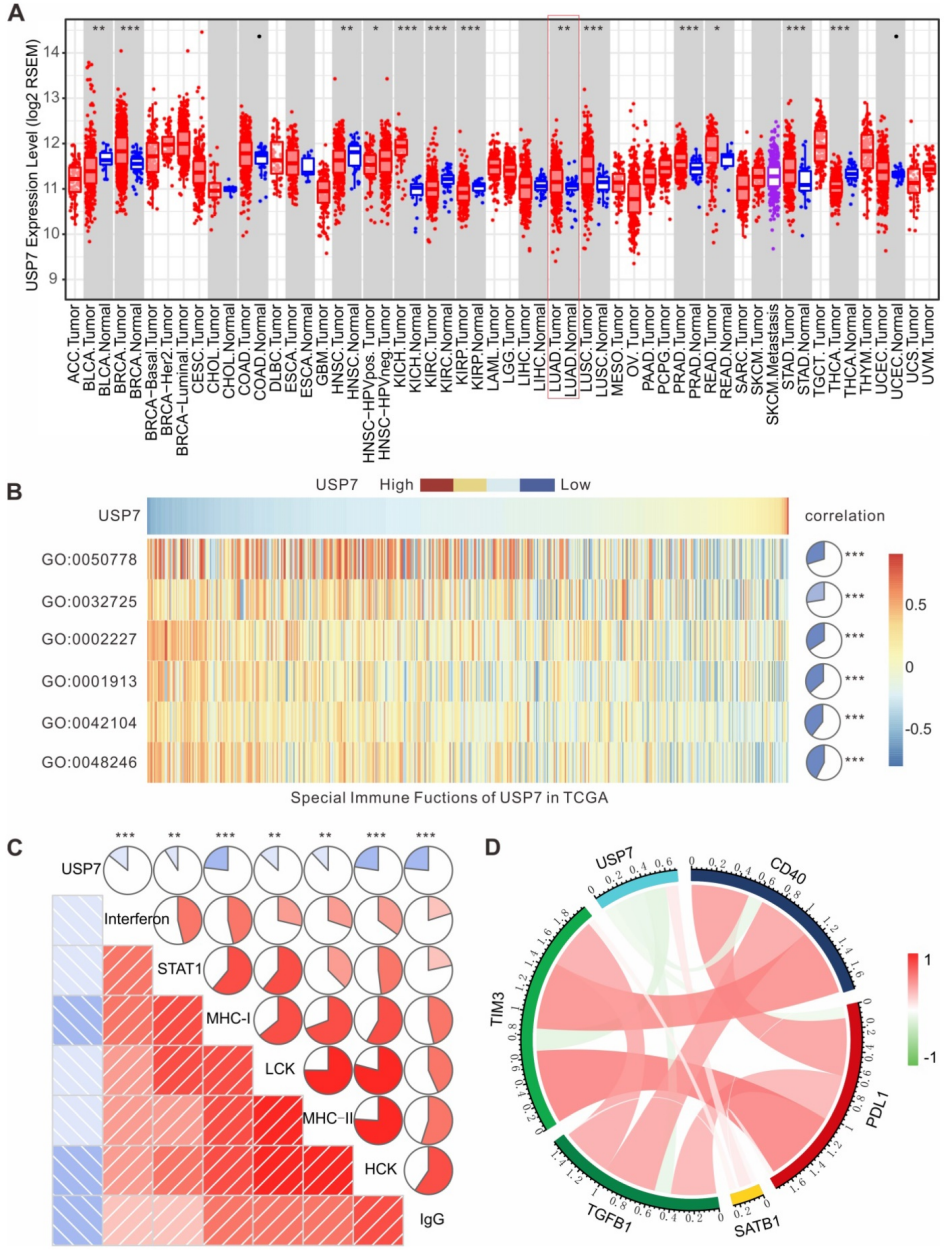
** USP7 expression in LUAD is negatively correlated with anti-tumor immunity in TCGA. (A)** USP7 mRNA expression levels in various human cancers (red), normal tissues (blue) and metastatic tissues (purple) from TCGA database. (**B**) The association between USP7 and innate and T cell-mediated adaptive anti-tumor immunity. GO:0048246, MΦ chemotaxis. GO:0042104, positive regulation of activated T cell proliferation. GO:0001913, T cell regulated toxicity. GO:0002227, innate immune response in mucosa. GO:0032725, positive regulation of granulocyte MΦ colony-stimulating factor production. GO:0050778, positive regulation of immune response. (**C**) The correlation between the USP7 expression and T cell immunity and inflammatory immunity. (**D**) Correlation of the members related to immune regulation in LUAD.

To further investigate the role of USP7 in the immune microenvironment of human lung adenocarcinoma (LUAD), we analyzed the results of transcriptome sequencing for 517 patients with LUAD in TCGA database. As shown in Figure 7A, mRNA expression of USP7 in LUAD is significantly higher than that in normal lung tissue, indicating that USP7 may be an oncoprotein in LUAD. Moreover, we performed gene set variation analysis (GSVA) to explore the relationship between the USP7 expression levels and immune responses in LUAD. We found that the high expression of USP7 was negatively correlated with MΦ chemotaxis (GO: 0048246), T cell activation and proliferation (GO: 0042104), T cell regulated toxicity (GO: 0001913), mucosal innate immune response (GO: 0002227), granulocyte macrophage colony-stimulating factor (GM-CSF) production (GO: 0032725), and immune response (GO: 0050778) (**Figure 7B**). Furthermore, we explored if the USP7 expression was correlated with T cell immunity and inflammatory immunity by analyzing 7 sets of collective genes, according to the method reported by Rody et al. 41. As shown in **Figure 7C**, the expression of USP7 was negatively correlated with HCK, LCK, MHC-I, MHC-II, STAT1, interferon, and IgG - indicating that USP7 may play a negative regulatory role in T cell immunity and inflammatory immunity. Finally, we analyzed the correlation between the expression of USP7 and molecules related to negative immune regulation (TIM-3, TGFB, PD-L1, *etc*.). As depicted in **Figure 7D**, the expression of USP7 was negatively correlated with PD-L1, TIM-3, TGF β, and CD40; while there was a positive correlation with SATB1. Altogether, USP7 appears to play a negative regulatory role in anti-tumor immunity in human LUAD.

## Discussion

In recent years, anti-tumor strategies aimed at reshaping the TME have attracted a lot of attention 46. TAMs are one of the most important components of TME - they can synthesize and secrete anti-inflammatory factors, inhibit anti-tumor immune responses, and promote tumor growth - thereby contributing to tumor progression and metastasis with strong clinical evidence 47. Approaches for reprogramming of TAMs are being actively pursued, and improved strategies are required for precise and efficient directing of MΦs towards the anti-tumor activity. Ubiquitinating/Deubiquitinating system is an important and classic regulatory tool for cells to govern degradation and homeostasis of proteins produced by environmental stimuli, and it is closely linked to tumor occurrence, development and inflammatory immune regulation 23. Few studies have reported the role of DUBs in regulating TAMs. We screened the 51 mRNAs of common proteins in the DUB system for their expression in M1 and M2 MΦs, and found that the mRNA of USP7 was the most differentially expressed one between M2 and M1 MΦs. We further verified that the abundance of translated USP7 protein in M2 MΦs was significantly higher than in M1 MΦs. Moreover, differential expression of USP7 mRNA and the corresponding protein was verified in M2 and M1 MΦs induced from BMDMs, inspiring us to further investigate the role of USP7 in the regulation of MΦ activity.

Our study showed that targeting USP7 could significantly inhibit the phenotype and function of M2 MΦs. Meanwhile, *in vivo* results showed that targeting USP7 could significantly inhibit the growth of Lewis lung cancer in mice, reprogramming TAMs to M1 MΦs, and promoting TME CD8^+^T cell activation. Moreover, we found that P5091 (an inhibitor of USP7) was able to activate the systemic anti-tumor T cell immune response. Several studies were focused on the relationship between USP7 and the immune system. Among other things, they have reported that targeted inhibition of USP7 can inhibit the function of Treg cells by promoting the ubiquitin degradation of Foxp3 39. We speculate that the reason why the proportion of Treg cells in the TME and the spleen does not decrease in this study may be the relatively lower dose of P5091, as well as sensitivity differences between TAMs and Treg cells.

USP7 plays an important role in tumorigenesis and tumor development through interaction with the protein MDM2 48. Targeting USP7 has an inhibitory effect on various tumors 49-52. In this study, we found that USP7 inhibitor P5091 significantly inhibited the growth of Lewis tumor in mice. To identify whether the P5091-mediated tumor therapy efficacy was dependent on reprogramming TAMs to activate anti-tumor immune responses, we depleted TAMs by chlorophosphate liposomes and found that the inhibitory effect of P5091 on tumor growth and the effect on CTL activation were partially attenuated, indicating that the anti-tumor activity of P5091 depended on the function of MΦs. According to **Figure S11**, P5091 inhibited the growth of Lewis cells with an IC50 of about 12 μM. Considering that the anti-tumor activity of P5091 has not completely disappeared after the clearance of MΦs (**Figure 4C**), we speculate that the inhibitory effect of targeting USP7 on the growth of Lewis tumor in mice may be based on its dual effects on tumor cells and TAMs, in which remodeling of TAMs might play a major role.

Furthermore, we sorted TAMs from mice in the control group and P5091-treated mice by flow cytometry and RNA sequencing analysis to elucidate the molecular mechanism of remodeling tumor TAMs by USP7 targeting. KEGG enrichment analysis of differentially expressed genes in the two groups showed that MAPK pathway was significantly enriched. Related studies have reported that MAPK pathway is widely involved in the inflammatory activation of MΦs and reprogramming TAMs towards the M1 phenotype 16, 53. Western blotting also confirmed that USP7-targeted inhibitors can significantly promote activation of the JNK/ERK/p38 MAPK pathway in MΦs. Based on pathway rescue experiments, targeted inhibition of USP7 activated the p38 MAPK pathway, leading to TAM reprogramming.

Targeting USP7 with P5091 up-regulated the expression of PD-L1 in TME, indicating that the combination with PD-1 monoclonal antibody therapy may improve the anti-tumor effect of the treatment. Indeed, the combination of P5091 with PD-1 monoclonal antibody treatment led to slower tumor growth and longer survival time than either single-drug treatment. However, the mechanism of PD-L1 expression up-regulation by targeting USP7 in Lewis tumor cells is not clear and we plan to further explore it in the future.

In addition, we further verified the relationship between USP7 and the immune system in human LUAD specimens. By analyzing the sequencing data of 517 cases of LUAD in TCGA database, we found the expression of USP7 in LUAD was significantly higher than that in normal lung tissue, and the high expression of USP7 was negatively correlated with innate immunity and specific T cell immune activation. At the same time, the expression of USP7 was negatively correlated with molecules related to negative immune regulation, such as PD-L1, Tim3, and TGF-β, indicating that USP7 may negatively regulate the expression of PD-L1 in tumor cells. In our study, the expression of PD-L1 in tumor cells and TME was up-regulated after the application of P5091, which further verified the hypothesis, but further research is needed. Moreover, considering that many other DUBs mRNA were found to be differentially expressed between M1 and M2 MΦs (Figure 1A), further investigation into other DUB targets is warranted in future studies.

## Conclusions

To the best of our knowledge, this study is the first to show that USP7 plays an important role in reprogramming TAMs in lung cancer. Targeted inhibition of USP7 reprogrammed TAMs to M1 MΦs by activating p38 MAPK pathway, and then activated the anti-tumor effect mediated by CTL, and finally inhibited tumor growth. However, targeted inhibition of USP7 can increase the expression of PD-L1 in tumor microenvironment, and further combination with PD-1 monoclonal antibody can exert synergistic antitumor effect. In general, targeting USP7, combined with the PD-1 monoclonal antibody, may provide a new avenue for clinical treatments of lung cancer.

## Supplementary Material

Supplementary figures and tables.

## Figures and Tables

**Scheme 1:**
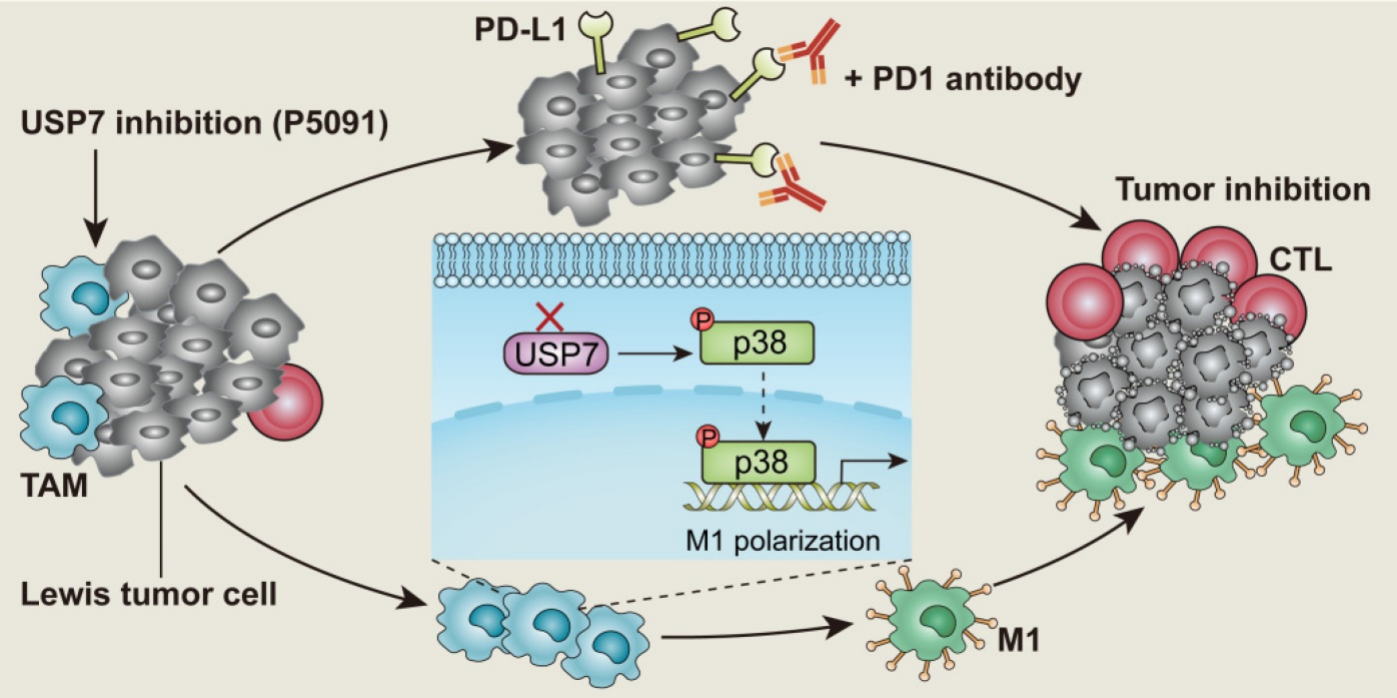
Targeted inhibition of USP7 modulates anti-tumor immune response by reprogramming tumor-associated macrophages in lung cancer.
