# Supplementary material for: USP7 targeting modulates anti-tumor immune response by reprogramming Tumor-associated Macrophages in Lung Cancer
Source: Theranostics. 2020 Jul 23;10(20):9332–47. doi: 10.7150/thno.47137 (PMC7415808; doi:10.7150/thno.47137)
Supplement: Supplementary file 1 — Supplementary figures and tables. [file thnov10p9332s1.pdf]

USP7 targeting modulates anti-tumor immune response by reprogramming tumor-associated macrophages in lung cancer

Xiaomeng Dai[†1], *Lisen Lu*[†1], *Suke Deng*[†], *Jingshu Meng*[†], *Chao Wan*[†], *Jing Huang*[†], *Yajie Sun*[†], *Yan Hu*[†], *Bian Wu*[†], *Gang Wu*[†], *Jonathan F. Lovell*[‡], *Honglin Jin*[†*], *Kunyu Yang*[†*]

[†]Cancer Center, Union Hospital, Tongji Medical College, Huazhong University of Science and Technology, Wuhan 430022, China

[‡]Department of Biomedical Engineering, University at Buffalo, State University of New York. Buffalo, New York 14260, USA

[1]These authors contributed equally to this work.

[*]Correspondence: Cancer Center, Union Hospital, Tongji Medical College, Huazhong University of Science and Technology, Wuhan 430022, China. Fax: +86-27-65650733; Tel: +86-27-85873100.

Email: Kunyu Yang, yangkunyu@medmail.com.cn; Honglin Jin, jin@hust.edu.cn

Supplementary Table S1. RT-PCR primer information

Gene	Forward (5' to 3')	Reverse (5' to 3')
USP1	AGATGTGTGAACTAGGTAAGCC	ATACTTCATCGTCGTAGTTCCC
USP3	GTTTGCTCCTTAAGAGATTGCC	TCTCAGTGGGAATTGAACGTAT
USP4	CAGGCAACAAAGAAGTTTGACT	CCGCCAGTATCTGTTATAGGAG
USP5	GAAAGCTGTGTACTATACGGGA	GGGTTTGCAAAATCTGGATCAT
USP7	TCGTCGCACATTGAGACGG	CTTGTCGGCATGGTTGGGAAT
USP8	GGACCCTTGGTCATCGACAG	CACCAGCTTTGAACAGCAGATA
USP9x	TCCAACAGAATCAGACTTCATCG	TGGAAATGCAGGTTCCTCATCT
USP10	CTGAAGCCGTTGAAAAAGATGAG	TCAGCCTCTGCGTTAGAGTTG
USP11	GCAGAACCATAAACGACGAAAT	CACAGATCTGAGATATTGCCCT
USP12	CAGTCTCCAAATTCGCCTCCA	GTGCTCGTTGACCGGAAACT
USP13	CAGTCTGCCCTTGGCATTTAC	CCTCTTCTCTTAGGGAGCACAG
USP14	ACCTCCAATGGTGTTCAAAGC	CATCCTTCAGGGTTCCTCCTT
USP15	TCAGCCATTCAGTGTTTGAGC	TTTCACCTCTCATTCCTAAGGGA
USP16	TGGCTCCTTTTTGTACCCTTAA	GTACCACTGTGTTCAACAACTC
USP18	CAGGAGTCCCTGATTTGCGTG	CAGAGGCTTTGCGTCCTTATC
USP19	TGGAGATGCTAGGAGAGTGTC	CGCAGCTTAACAATCACCTCAT
USP20	TGGACTGCATAGGGGAGGTG	GGCAAGCCCATAGGTTAGGTC
USP21	AACTCCATGTTACGACCTTTGC	AAGGGGACCTCTAGGACGAGA
USP22	TCTTTCTGTCGGATAGGCACC	GCCCTGAGTAAAACTCCTGGA
USP24	GCTGGAAAGCCGCGTTTTG	CAAGTCTGGCTAAGTAGGTGGA
USP25	TCTCGAAACCCCTATGACAGAA	TGAATAACTGCACTAAACCAGCA
USP27x	GACATTGAGCAAATTGCCAAAGA	AAGTCCCGGCACTGAACAC
USP28	GGGTCCGAGAAGGAAAGCC	CACGGAACGATCCGAAGGAAG
USP29	GCTGGGTGATAATGTTACAGGC	TGAGTAAGGATGTGTCGTCTCT
USP30	CCTCACCCCACGACCAATC	GAGAAAGGCTGTCAAAGGTGT
USP31	TTCTTTGCCAATTCCTCTACCC	CACAGCCACACCGATCCTC
USP32	GTCCCAGATACACTCAGGAAGT	AGCGAGAGAAGGTAAAAGCATC
USP33	GGACCACAGCACCATACACTC	GCTGCAAGCATAACACCATACT
USP34	CTGGTTGCCTATGAAGGCTTG	AGCTTGATGCAGTTTTTCGACA
USP36	CCAACAGCGGCAATGCTATC	CATCGCATCAATGGTGTACCG
USP37	AGTCAGCCTGCTCGTTCACTA	AAGTCAACATTAGGCGGCTTT
USP38	GCCCCTCAAGCGGATGATT	GGGTCGTCAGGTCAAACATGG
USP39	GTCACTGCCCGTACTTGGATA	GTATGCGTTGATGTGCGAGAG
USP40	TGACCGACTGGTTAAAGCAGC	GCTAGTATCCTTGTAGCGTTCAC
USP42	AGGCGGTCTCACCTGAAGA	CACTGGCCCTAATGGAAGTGT
USP45	ATGCGGGTAAAAGATCCATCAAA	ACGTTAGACCTACAGCAATGTCA
USP46	ATGACTGTCCGAAACATCGCC	TTGACCAATCCGAAGTAGTGTTC
USP47	GATGTGATTCCCTTGGATTGCT	AACCCCATTGGTGTATCTTCTTC
USP48	CAGAGGAAACCCGAATTGCTT	GTGGCTCCCAGGTTAGTCAAG
USP49	AGTTCCGGGAATGTTTCCTGA	CTCCTTACTGACAACTCTGCG
BAP1	TGGGTCTGCCTATTGGCTTCT	AGCCCACTCAATGGTTTTCTTTT
UCHL3	TCAAGGACAAGATGTGACATCA	TTCGAAGTGCATCTTGTCTTTG

UCHL5	AGTGTACTACTGAACTGTACGCA	CACTTGTCGAATCACATCTGAGT
OTUB1	TGATGGCAACTGCTTCTACCG	GTCCTCTTTACTCTTGGCAGAC
OTUB2	AACTCAGCAAAAGATTCACCTCG	TCATTTGGGGTCTGTAGCACA
OTUD7B	TGCTGTCCTGTCGGATTTTGT	TGGACTTGACGCAACTGTTCA
OTUD1	AGAGGCAGGACAAGTACCTGA	CCCGTACACAGTCTTGCTGAC
OTUD3	CTTCGTGGAAGATGACATTCCC	AAAGGGGCATTAAGCTGATGG
OTUD5	AGGACGGTGCCTGTCTATTTC	CATCAGATAGTCCATGCAATGCT
CYLD	ACCCTACTGGGAAGAACGGAT	CGGTCTTGGATGTACTGTCCTAT
ZRANB1	CCAGACTCTAGTGCAAGACCA	GGTCCTACGCTGGGATAAGC
Arg1	GGTTCTGGGAGGCCTATCTT	CACCTCCTCTGCTGTCTTCC
Chi3	CCCTGGGTCTCGAGGAAGCCC	GCAGCCTTGGAATGTCTTTCTCCAC
IFN-γ	GAGCCAGATTATCTCTTTCTACCT	GTTGTTGACCTCAAACTTGGC
IL-1	ACCCCAAAAGATGAAGGGCTG	TACTGCCTGCCTGAAGCTCT
IL-10	CAGAGAAGCATGGCCCAGA	TGCTCCACTGCCTTGCTCTTA
IL-12p35	GGAACTACACAAGAACGAGAG	AAGTCCTCATAGATGCTACCA
IL-12p40	TGGTTTGCCATCGTTTTGCTG	ACAGGTGAGGTTCACTGTTTCT
IL-6	GAGGATACCACTCCCAACAGACC	AAGTGCATCATCGTTGTTCATACA
TNF-α	CATCTTCTCAAAATTCGAGTGACAA	TGGGAGTAGACAAGGTACAACCC
VEGF	AAAAACGAAAGCGCAAGAAA	TTTCTCCGCTCTGAACAAGG
Nos2	CACCAAGCTGAACTTGAGCG	CCATAGGAAAAGACTGCACCG

Supplementary Table S2. The antibodies information

Antibody	Catalog number	Company
USP7	#4833	Cell Signaling Technology
JNK	#9252	Cell Signaling Technology
p-JNK	#4668	Cell Signaling Technology
ERK1/2	#4695	Cell Signaling Technology
p-ERK1/2	#4370	Cell Signaling Technology
P38	#8690	Cell Signaling Technology
p-p38	#4511	Cell Signaling Technology
β-actin	GB11001	Servicebio
GAPDH	GB11002	Servicebio
PD-L1	17952-1-AP	Proteintech

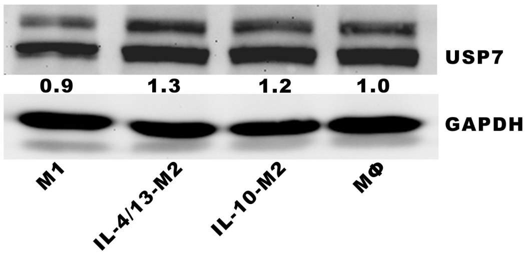

Figure S1. Western blotting showing the expression of USP7 in MΦ, M1, and M2 induced from ANA-1**.**

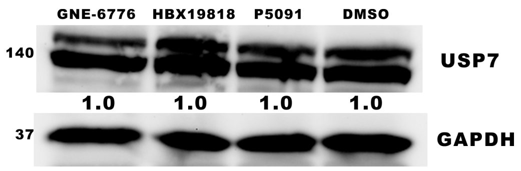

Figure S2. Western blotting showing the expression of USP7 in ANA-1 from various indicated treatments.

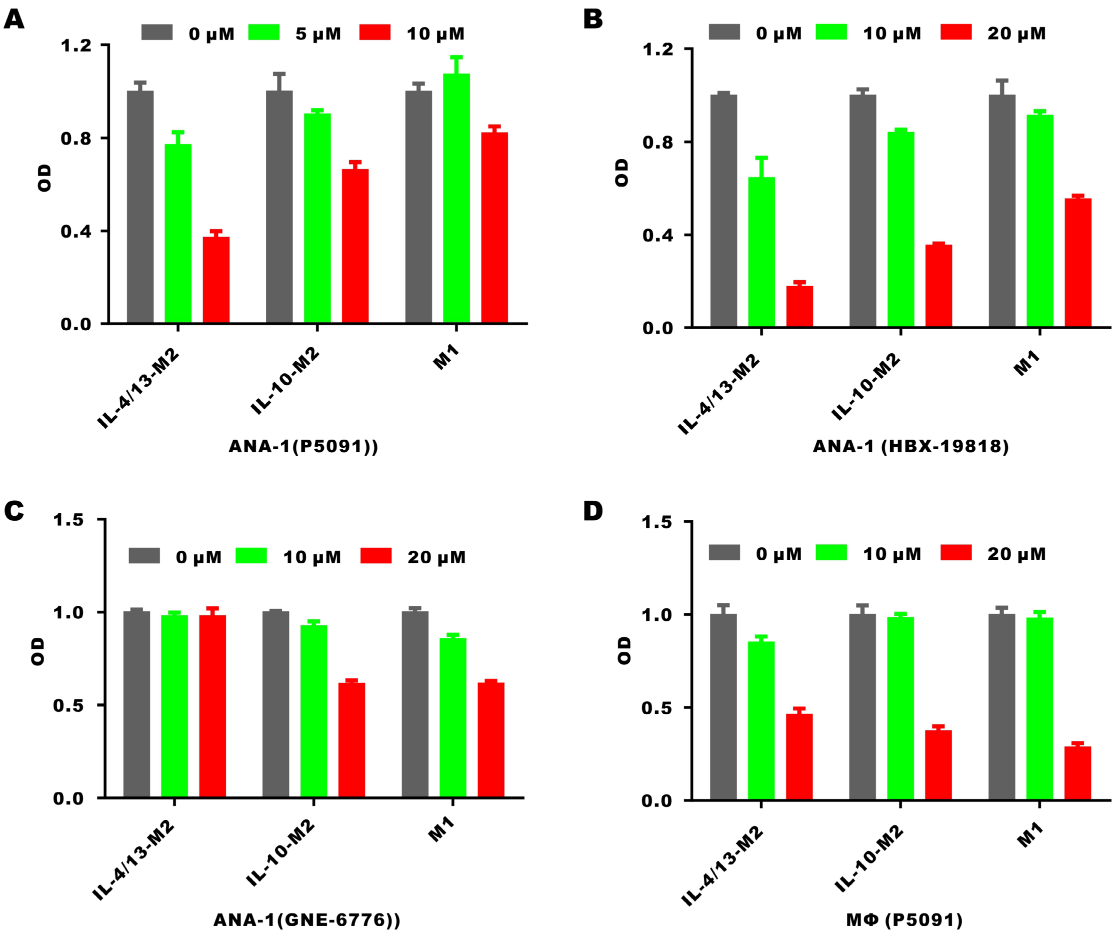

Figure S3. Cell viability detected by CCK-8 of M1 and M2 in the presence of USP7 inhibitors using indicated concentrations. (**A-C**) Effects of USP7 inhibitor P5091 (**A**), HBX19818 (**B**), and GNE-6776 (**C**) on the viability of M1, IL-4/13 M2, and IL-10 M2 induced from ANA-1. (**D**) Detection of the effect of P5091 on the viability of M1, IL-4/13 M2, and IL-10 M2 induced by BMDMs. Data are presented as the mean ± SEM (n = 3) for (A-D).

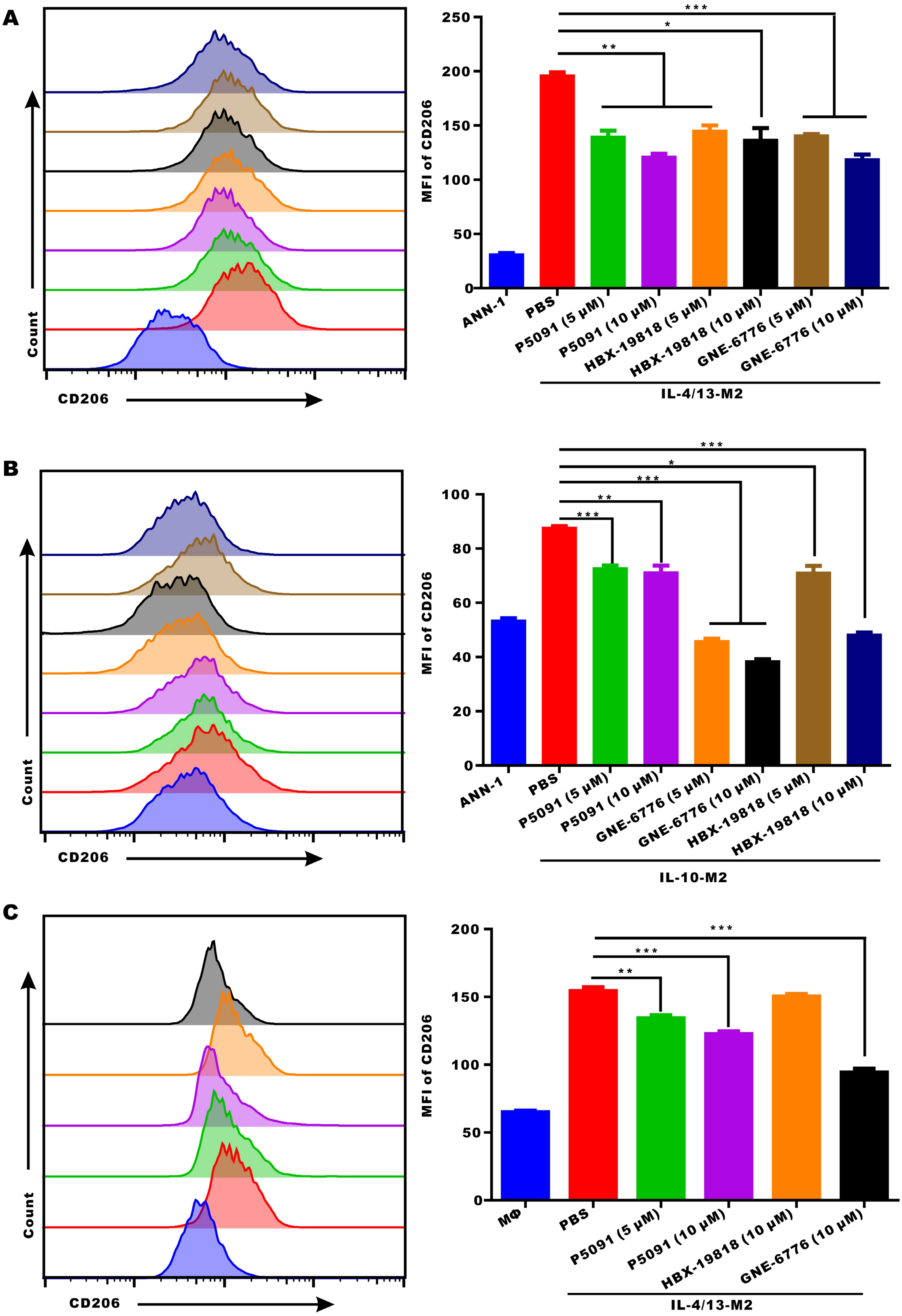

Figure S4. Flow cytometry analyses of the expression of CD206 in M2 MΦs in the presence of USP7 inhibitors or PBS. (**A-B**) Detection of the expression of CD206 in IL-4/13-M2 (**A**) and IL-10 M2 (**B**) induced from ANA-1 with indicated various USP7 inhibitors or PBS. (**C**) CD206 expression in IL-4/13-BMDM M2 treated with indicated various USP7 inhibitors or PBS was analyzed by flow cytometry. Data are presented as the

mean ± SEM (n = 3) for (A-C).

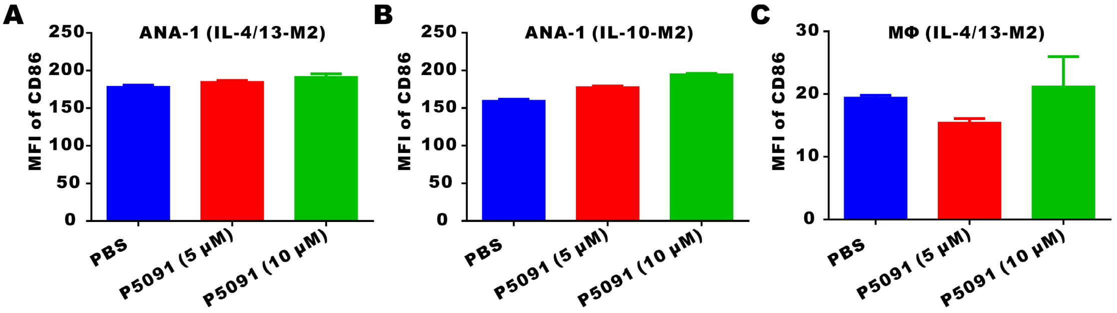

Figure S5. **Detection of the expression of CD86 in M2 MΦs in the presence of P5091 or PBS by flow cytometry.** (**A-B**) The expression of CD86 in IL-4/13 M2 (**A**) and IL-10 M2 (**B**) induced from ANA-1 was analyzed by flow cytometry in the presence of P5091 (5 μM and 10 μM) or PBS. (**C**) Quantification of CD86 in IL-4/13-BMDM M2 treated with indicated various treatments by flow cytometry. Data are presented as the mean ± SEM (n = 3) for (A-C).

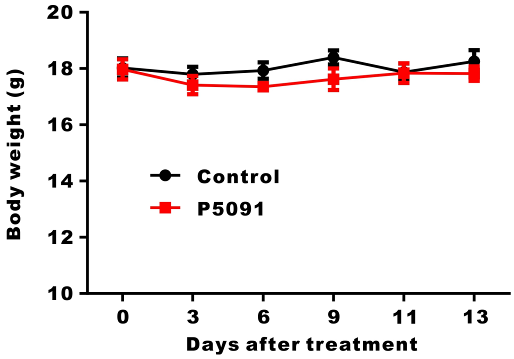

Figure S6. Measurement of the body weight in each group. Data are presented as the mean ± SEM (n = 6).

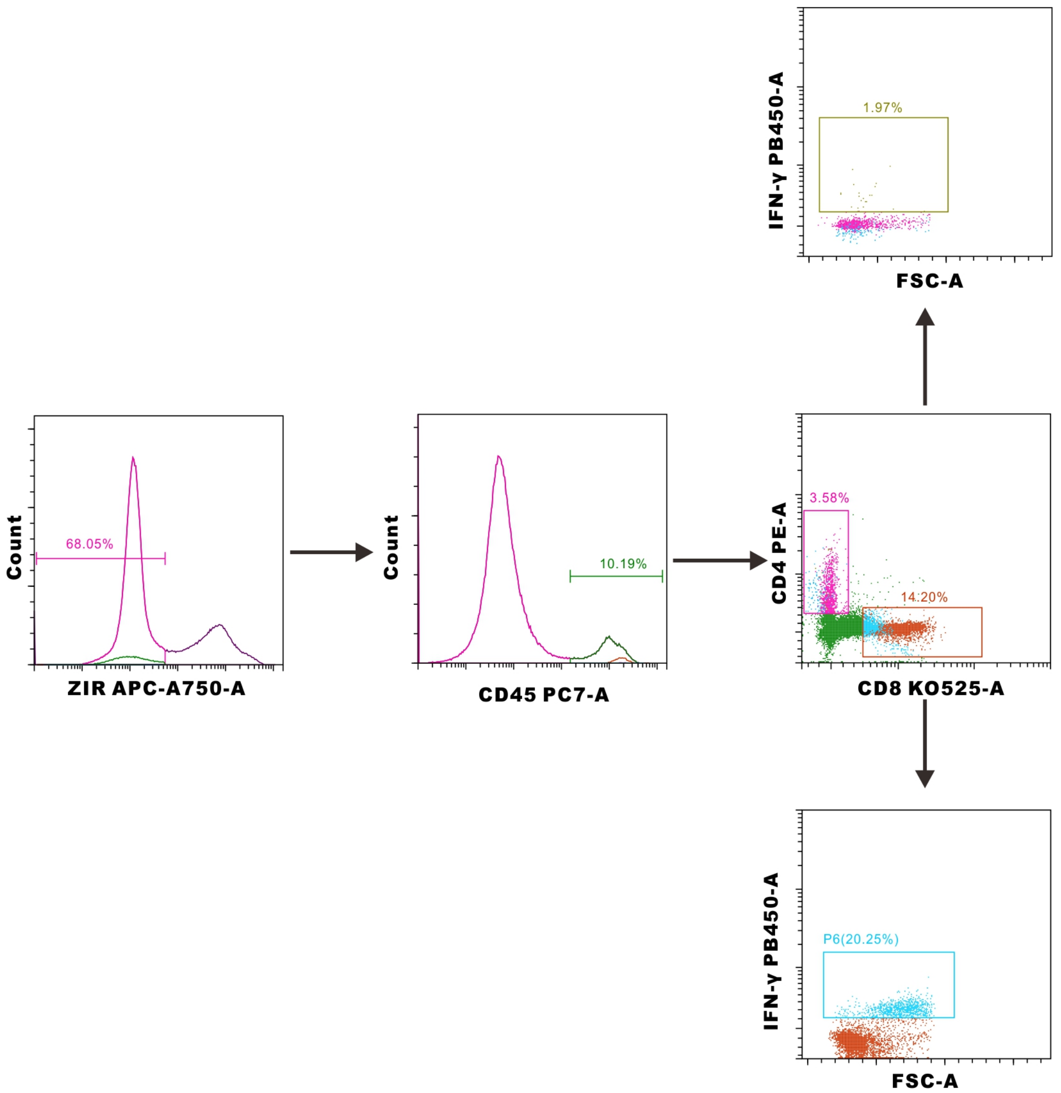

Figure S7. The gating strategy for the detection of Th1 cells and CTLs in TME by flow cytometry.

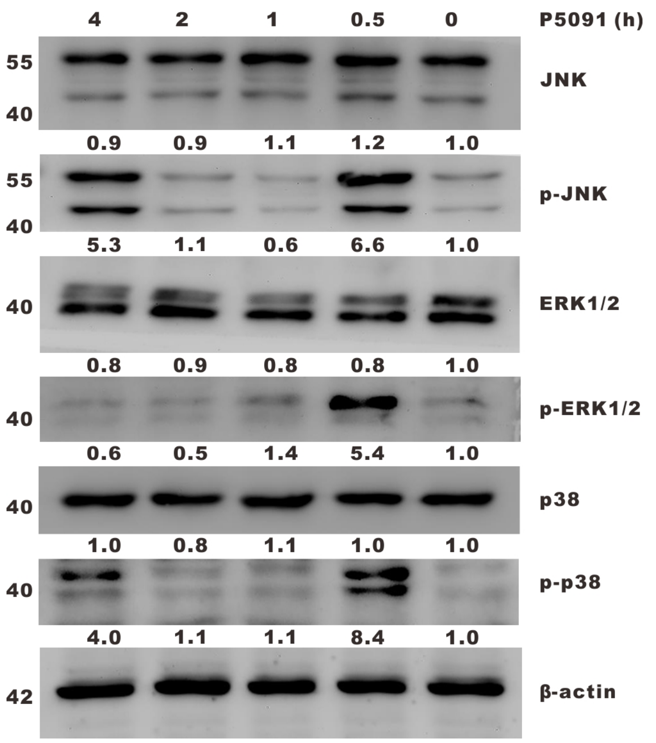

Figure S8. The expression of JNK, p-JNK, ERK1/2, p-ERK1/2, p38, p-p38 and β-actin in IL-4/13 M2 cells (IL-4/13-induced ANA-1) treated with P5091 (10 μM) at indicated time points by western blotting.

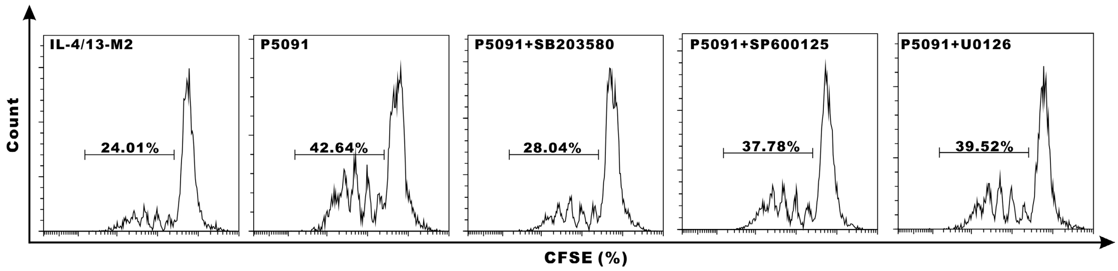

Figure S9. Flow cytometry analyses of CFSE expression on the CD8^{+} T cell membrane surface after various indicated treatments.

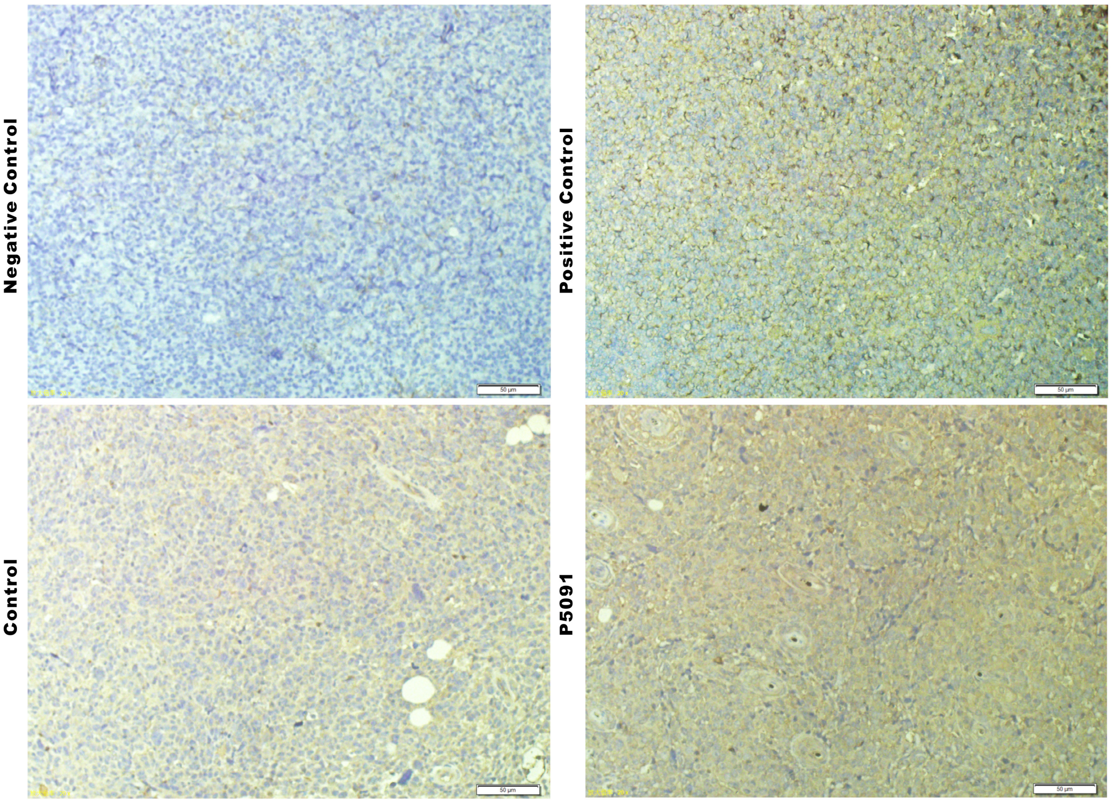

Figure S10. Immunohistochemical detection of PD-L1 expression in TME of P5091 and control groups.

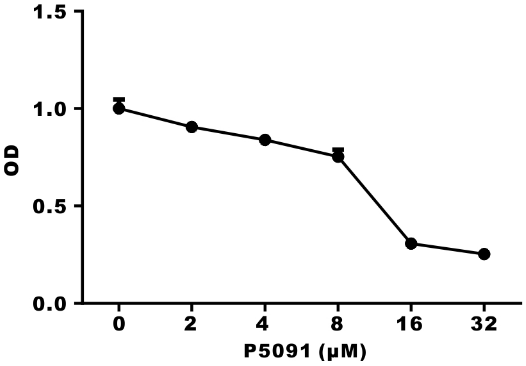

Figure S11. Cell viability measurement of Lewis cells treated with P5091 at indicated concentrations by CCK-8. Data are presented as the mean ± SEM (n = 3).
